# Selection and validation of reference genes for RT-qPCR analysis of different organs at various development stages in *Caragana intermedia*

**DOI:** 10.1515/biol-2022-0463

**Published:** 2022-09-14

**Authors:** Jinhua Liu, Chuang Yang, Mingzhu Bai, Feng Yan, Haiying Qin, Ruigang Wang, Yongqing Wan, Guojing Li

**Affiliations:** College of Life Sciences, Inner Mongolia Key Laboratory of Plant Stress Physiology and Molecular Biology, Inner Mongolia Agricultural University, Hohhot 010018, P.R. China; Inner Mongolia Enterprise Key Laboratory of Tree Breeding, Mengshu Ecological Construction Group Co., Ltd., Hohhot 011517, P.R. China; Inner Mongolia Engineering Research Center for Plant Gene Resources Mining and Molecular Breeding, Inner Mongolia Agricultural University, Hohhot 010021, P.R. China; Ordos Forestry and Grassland Development Center, Ordos 017010, P.R. China

**Keywords:** *Caragana intermedia*, development stage, RT-qPCR, reference gene

## Abstract

Reverse transcription quantitative PCR (RT-qPCR) is a technique widely used to investigate the expression of genes. An appropriate reference gene (RG) is essential for RT-qPCR analysis to obtain accurate and reliable results. *Caragana intermedia* plays an important role in afforestation as a bush. However, due to the lack of appropriate RGs, the research on development-related genes is limited. In this study, the selection for suitable RGs of different organs at various development stages to normalize the results of RT-qPCR about development-related genes was performed. To test the expression stability across all samples, we used the software algorithms such as geNorm, NormFinder, BestKeeper, and RefFinder to evaluate all the candidate RGs. Our results showed that *CiEF1α* was the most stable RG with little fluctuation among all samples. In addition, *CiGAPDH* in roots, *CiSKIP1* in stems and leaves, and *CiEF1α* in different organs were selected as the most stable RGs. To confirm the applicability of the most stable RGs, the relative expression of *CiWRKY17* was normalized using different candidate RGs. Taken together, our research laid a foundation for the study of development-related genes in *C. intermedia*.

## 1 Introduction

*Caragana intermedia*, commonly known as a bush, belongs to the legume family and is widely distributed in north and northwest China along with semi-fixed or fixed sand dunes, barren land, and loess hills. *C. intermedia* has high ecological value via playing an important role in afforestation [1]. To make the bush perform its role, it is essential to study the growth and development of *C. intermedia* to achieve the goal of cultivating healthy seedlings quickly and transferring them efficiently for afforestation.

Today, with the wide recognition of the important role of growth and development of plants, there have been many studies about development [2,3], and the research on development-relevant genes in *C. intermedia* has also made some progress [4]. In order to better uncover the function of these genes, it is pivotal to analyze their spatio-temporal expression [5]. Reverse transcription quantitative PCR (RT-qPCR) was often used to study the expression of genes because of its high throughput, specificity, and sensitivity [6–8], but its accuracy is difficult to guarantee due to changes in mRNA quantity and quality, and other reasons. Using relatively stable reference genes (RGs) can be a good solution to this problem. Therefore, it is necessary to select appropriate RGs.

Under ideal conditions, the RGs should be relatively stable, but some reports indicated that the expression of these so-called RGs could fluctuate in different organs and at various stages in plants [9–12]. For example, the commonly used RGs, such as Actin (*ACT*) [13], Beta-tubulin (*TUB*) [14], Elongation factor 1-α (*EF1α*) [10,15], and glyceraldehyde-3-phosphate dehydrogenase (*GAPDH*) were often chosen for normalization of RT-qPCR in plants, but *EF1α* showed no expression stability in the root of *Prunus* spp. [16], and *TUB* was one of the least stable genes in the cells elicited with MeJA of *Cichorium intybus* [17]. So it is quite necessary to screen RGs for their suitability for different experimental designs. Owing to no perfect method to evaluate the stability of RGs, four different analytical software, such as geNorm [18], NormFinder [19], BestKeeper [20], and RefFinder [23], were usually used to identify the suitable RGs.

Previous studies showed that the RGs of *C. intermedia* [21] had been screened under various abiotic stresses. However, these RGs were not screened in different organs or at various development stages, so this study aims to select relatively stable RGs for the reference of the development-related genes of *C. intermedia*. First, we selected 11 commonly used RGs, such as *CiACT*, *CiCAP* (Cyclase-associated protein), *CiEF1a*, *CiGAPDH*, *CiSKIP1/SKIP5-1/SKIP5-2* (F-box proteins), *CiTUA* (Alpha-tubulin), *CiTUB/TUB3* (Beta-tubulins), and *CiUBQ* (Ubiquitin), which were proved to be relatively stable in its homologous species, *C. korshinskii* [22], and the expression level of these RGs were analyzed among samples from different organs or at various development stages by RT-qPCR. Next, all these selected RGs were ranked using geNorm, NormFinder, BestKeeper, and RefFinder software. Finally, *CiWRKY17*, which had been proved to express in different organs, might be involved in the growth and development of *C. intermedia* [4], was selected to validate these RGs. The above results will provide the most appropriate RGs for further study of development-related genes in *C. intermedia*.

## 2 Materials and methods

### 2.1 Collection of plant materials

**Figure 1:**
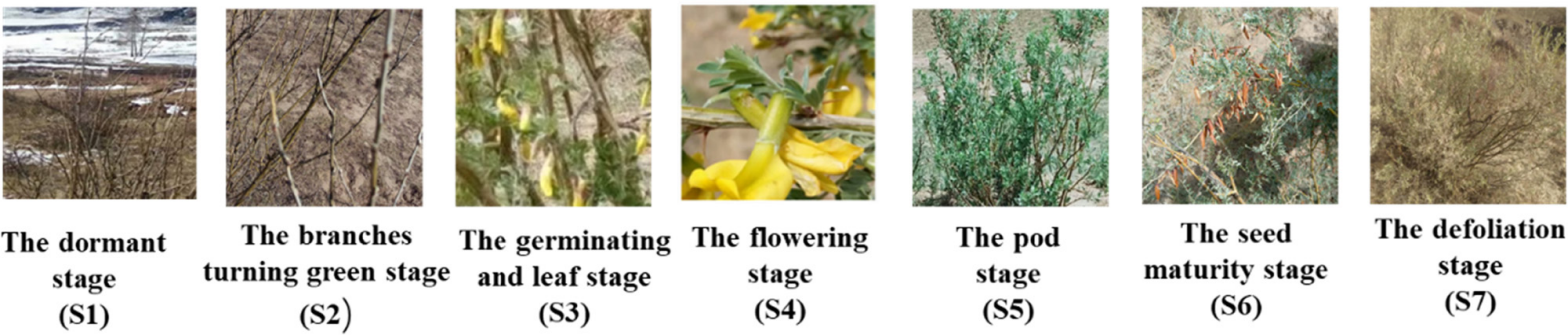
Different developmental stages of the samples.

The samples were collected in the field located in Liangcheng County, Ulanchabu City, Inner Mongolia, China, with north latitude 41°23′ and an east longitude 111°41′. Totally 69 samples from seven different stages of development (Figure 1), and seven organs, including root, stem, leaf, bud, flower, young pods, and young seeds, were collected as indicated in Figure 1 and Table 1. Each sample was taken from an even mixture of the same tissues from three different plants, and three biological replicates were performed. All samples were immediately frozen in liquid nitrogen for RNA extraction in the follow-up steps.

**Table 1:** The number of samples of different organs

	Stage	Root	Stem	Leaf	Bud	Flower	Pod	Seed
The samples	S1	3	3	—	—	—	—	—
	S2	3	3	—	—	—	—	—
	S3	3	3	3	3	—	—	—
	S4	3	3	3	—	3	—	—
	S5	3	3	3	—	—	3	—
	S6	3	3	3	—	—	—	3
	S7	3	3	3	—	—	—	—

### 2.2 RNA isolation and cDNA synthesis

RNA extraction and reverse transcription were performed using TaKaRa MiniBEST Plant RNA Extraction Kit and TaKaRa PrimeScriptTMRT reagent Kit with gDNA Eraser, respectively. RNA was measured using an ultraviolet spectrophotometer (Model DU800), and the ratio of A260 nm/A280 nm and A260 nm/A230 nm was calculated to check the purity and the concentration (μg/mL) of the extracted RNA. RNA integrity was assessed using electrophoresis of the extracted RNA on a 1.0% (w/v) agarose gel. Any RNA sample with an A260/A280 ratio between 1.8 and 2.2 and an A260/A230 ratio greater than 2.0 was used for subsequent experiments. One microgram of total RNA from each sample was used to synthesize the first-strand cDNAs using the above-mentioned reverse transcription Kit according to the manufacturer’s instructions. The cDNA acquired by reverse transcription was then diluted to 16-fold and used as the template for RT-qPCR.

### 2.3 Primer and RT-qPCR

The sequences for the primers of the selected 11 RGs were obtained from our previously published article [22] and are listed in Table S1. The primers’ sequence, amplification efficiency, the regression coefficient, *R* ^2^ value, and the melting curve are also listed in Table S1 or in Figure S1. The cDNA was amplified using TB Green qPCR Master Mix (TaKaRa) with a Roche LightCycler 480 system. The thermal cycling program was 95°C for 60 s, followed by 40 cycles of 95°C for 5 s, 60°C for 30 s, and 72°C for 15 s. Each RT-qPCR reaction was performed with three technical replicates. The Ct values, which took the mean value of three technical replicates, were pooled for the RG evaluation.

### 2.4 Data analysis

Stability analysis of the RGs was assessed using the Excel-based geNorm [18], NormFinder [19], BestKeeper [20], and RefFinder [23], which were the most widely used software to screen RG by RT-qPCR.

For geNorm and NormFinder algorithms, the raw Ct values from each sample were converted into relative quantity (RQ) values using the formula 2^−ΔCt^ (ΔCt = each Ct value − the minimum Ct value) [24]. The geNorm program first calculated an expression stability *M*-value for each gene, and *M*-values below 1.5 were supposed to be stably expressed, and a lower *M*-value indicated a more stable expression [18]. Moreover, the value of “*n*” was the optimal number of RGs when the pairwise value of variation (*V*_*n*_/*V*_*n*+1_) was below a cutoff value of 0.15 [18]. NormFinder was used to assess the stability of RGs based on the ANOVA model [19].

For BestKeeper and RefFinder programs, the raw Ct values were directly analyzed. BestKeeper examined the ranking of RGs based on the calculation of the variance and the standard deviation (SD) for each gene. Any gene with an SD value less than 1.0 was recommended as a gene with stable expression [20]. RefFinder, a user-friendly and web-based comprehensive tool, was developed for selecting RGs, and integrated the currently available computational programs to compare the rank of the tested RGs [23].

### 2.5 Validation of RGs

*CiWRKY17* was selected to verify the most stable RG, the most stable RG combination, and the worst RG by RT-qPCR. Each qPCR reaction was performed with three technical replicates. The experiment was repeated three times (with three biological replicates), and the results were consistent. Finally, the data were calculated using the 2^−ΔΔCt^ method.

## 3 Results

### 3.1 Expression profiles of the RGs

**Figure 2:**
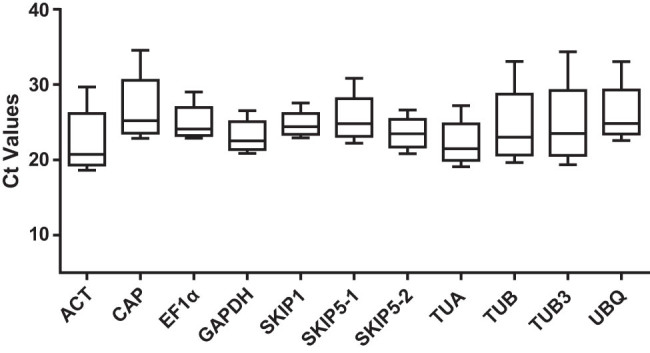
Distribution of the Ct values of the 11 RGs among all samples: the box plot graph shows the maxima, minima, medians, and the 25th/75th percentile.

The expression level of the 11 RGs was analyzed across all samples via determination of the Ct value, and lower the Ct value the higher the gene expression level was. The Ct value of these genes ranged from 18 to 34 in all studied samples, with most of them between 20 and 26, and the smaller the range of the Ct value the more stable the RG was (Figure 2).

To evaluate the relative stability value of RGs, we took the average Ct value and Ct value range. On the one hand, *CiACT* (with an average Ct of 21.15) showed the highest expression level, whereas *CiSKIP5-1* (with an average Ct of 24.9), *UBQ* (with an average Ct of 24.9), and *CiCAP* (with an average Ct of 25.2) showed relatively low expression level (Figure 2). On the other hand, the six genes with the minimum Ct value range were *CiSKIP1* (4.61), *CiGAPDH* (5.67), *CiSKIP5-2* (5.83), *CiEF1α* (6.14), *CiTUA* (8.12), and *CiSKIP5-1* (8.57) (Figure 2). In brief, based on both the higher expression level and the lower Ct value range, *CiSKIP1*, *CiGAPDH*, *CiSKIP5-2*, and *CiEF1α* were more appropriate as RGs.

### 3.2 Stability analysis by geNorm

**Figure 3:**
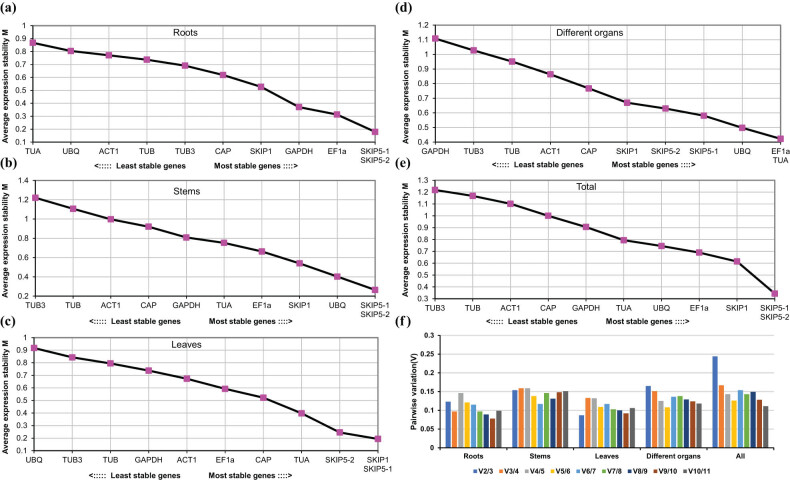
Expression stability measurement (*M*) and pairwise variation (*V*) analysis of the 11 selected RGs in samples from different organs or at various stages using geNorm: (a) roots; (b) stems; and (c) leaves from different developmental stages; (d) different organs (including buds, flowers, pods, roots, stems, leaves, and seeds samples), buds collected from the stage of S3, flowers collected from S4, pods collected from S5, and roots, stems, leaves, and seeds collected from S6; (e) all samples from different developmental stages; (a–e) represent all samples containing three biological replicates; and (f) pairwise variation (*V*) analysis.

According to geNorm analysis, the *M*-values of all tested RGs were below 1.5, indicating that they were relatively stable, and the lowest *M*-value indicated the highest stability [18]. Among all of the tested samples (Figure 3e), *CiSKIP1* was found to be the stable RGs successively, while *CiSKIP5-2* and *CiSKIP5-1* represented the most stable RG combination. On the contrary, *CiTUB3* was found to be the most inappropriate RG based on their fluctuating expression levels. In addition, these screening results of RGs varied in different organ samples (Figure 3a–d).

The optimal number of RGs for normalizing RT-qPCR data was determined by calculating the pairwise variations *V*_*n*_/*V*_*n*+1_ in the geNorm program, and when the value of *V*_*n*_/*V*_*n*+1_ is less than 0.15, the *n*-value is the optimal number of RGs [18]. As shown in Figure 3f, since the values of *V*2/3 and *V*3/4 were greater than 0.15 from left to right, and the value of *V*4/5 was below the cutoff value of 0.15, these four RGs were ideal for normalizing RT-qPCR data in all samples.

### 3.3 Stability analysis by NormFinder

**Figure 4:**
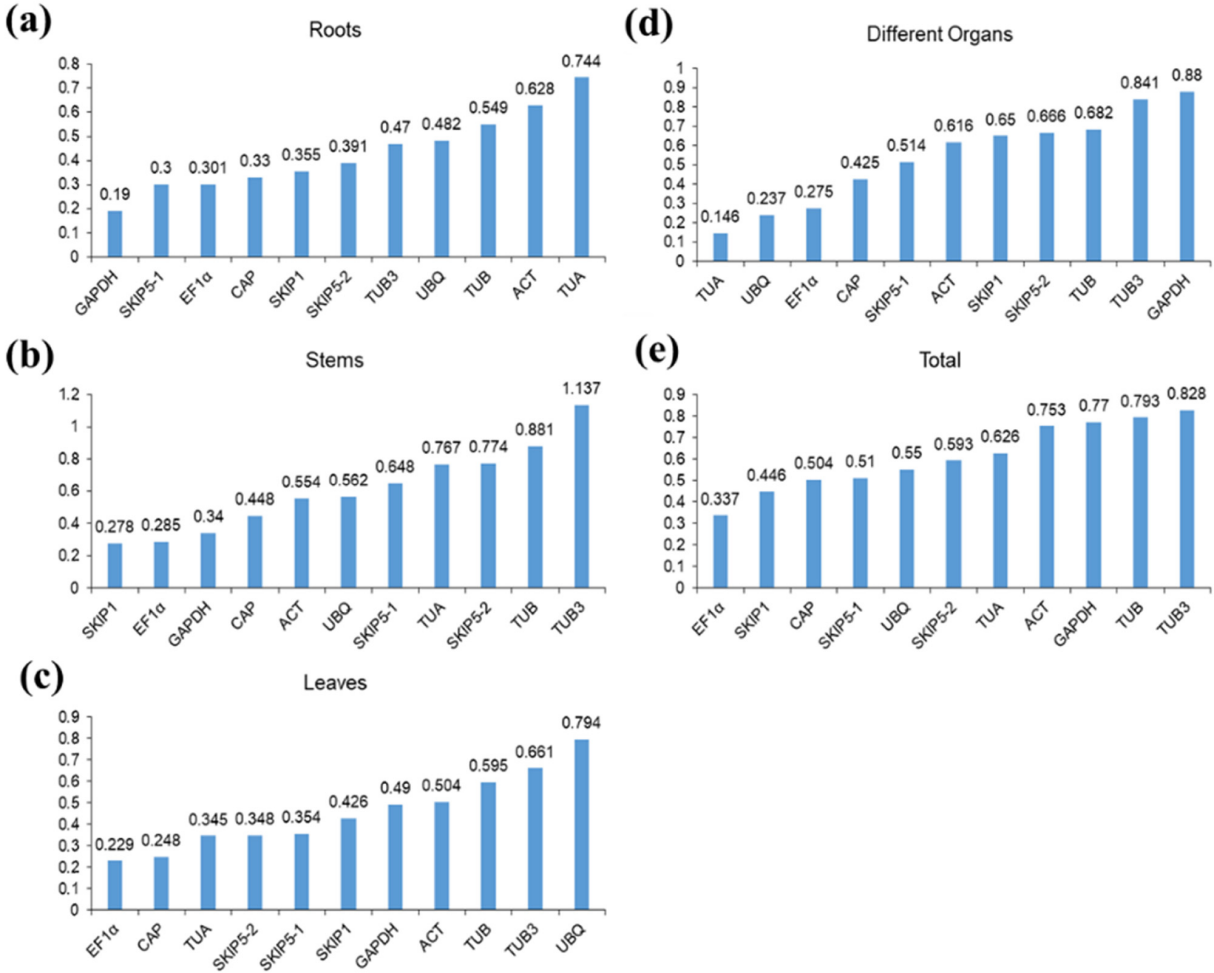
Stability value of the 11 RGs calculated by NormFinder: (a) roots; (b) stems; and (c) leaves from different developmental stages; (d) different organs (including buds, flowers, pods, roots, stems, leaves, and seeds samples), buds collected from the stage of S3, flowers collected from S4, pods collected from S5, and roots, stems, leaves, and seeds collected from S6; (e) all samples from different developmental stages; (a–e) represent all samples containing three biological replicates.

The NormFinder was similar to geNorm in algorithm to calculate different *M*-values of RGs. The difference was that NormFinder selected only one optimal gene, while geNorm selected two or more genes. The results calculated with NormFinder (Figure 4) showed that *CiEF1α* was the most stable gene in all the tested samples, while *CiTUB3* was considered to be a weakly stable gene, and there were some differences in different organ samples.

### 3.4 Stability analysis by BestKeeper

**Figure 5:**
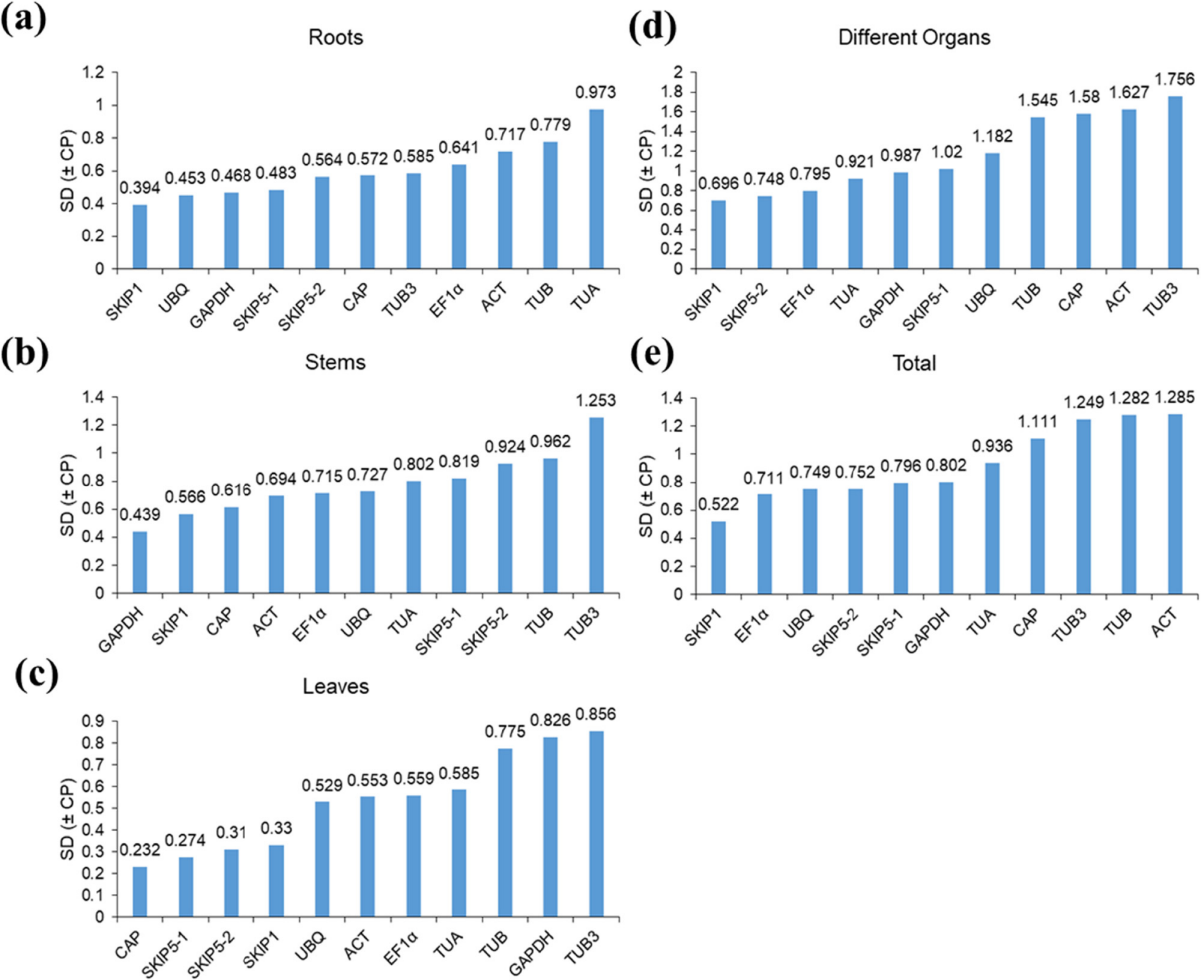
Stability value of the 11 RGs calculated by BestKeeper: (a) roots; (b) stems; and (c) leaves from different developmental stages; (d) different organs (including buds, flowers, pods, roots, stems, leaves, and seeds samples), buds collected from the stage of S3, flowers collected from S4, pods collected from S5, and roots, stems, leaves, and seeds collected from S6; (e) all samples from different developmental stages; (a–e) represent all samples containing three biological replicates.

BestKeeper examined the ranking of RGs based on the calculation of the SD value for each gene. Any gene with an SD value less than 1.0 was recommended as a gene with stable expression, and the lowest SD value was the highest stability of a gene. According to the above principles, the result showed *CiSKIP1* was the most stable gene, and *CiACT* was the least stable gene in all tested samples, and the results differed in different organ samples (Figure 5a–c).

### 3.5 Comprehensive stability analysis of RGs by RefFinder

**Figure 6:**
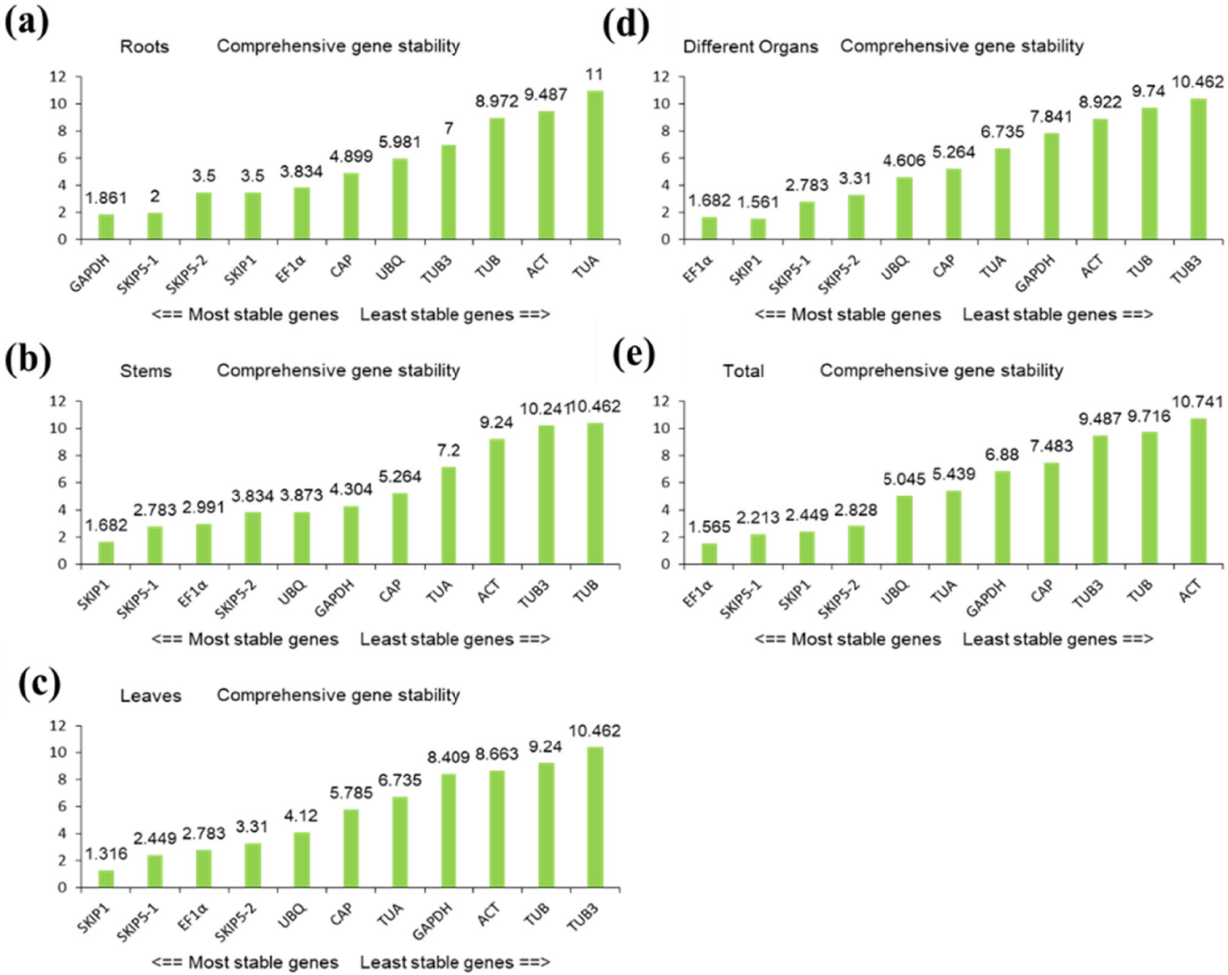
The comprehensive ranking of the 11 RGs by RefFinder: (a) roots; (b) stems; and (c) leaves from different developmental stages; (d) different organs (including buds, flowers, pods, roots, stems, leaves, and seeds samples), buds collected from the stage of S3, flowers collected from S4, pods collected from S5, and roots, stems, leaves, and seeds collected from S6; (e) all samples from different developmental stages; (a–e) represent all samples containing three biological replicates.

In order to comprehensively evaluate the stability of RGs, we made a comprehensive ranking by RefFinder to get the most stable RGs. As shown in Figure 6, *CiEF1α* was the most stable RGs with little fluctuation; moreover, *CiACT* was the least stable RGs due to large expression of fluctuations in all samples. Similarly, *CiGAPDH* in roots, *CiSKIP1* in stems and leaves, and *CiEF1α* in different organs were found to be the most stable RGs; *CiTUA* in roots and *CiTUB3* in stems, leaves, and different organs were not suitable to be used as RGs.

### 3.6 Verification of the selected RGs

**Figure 7:**
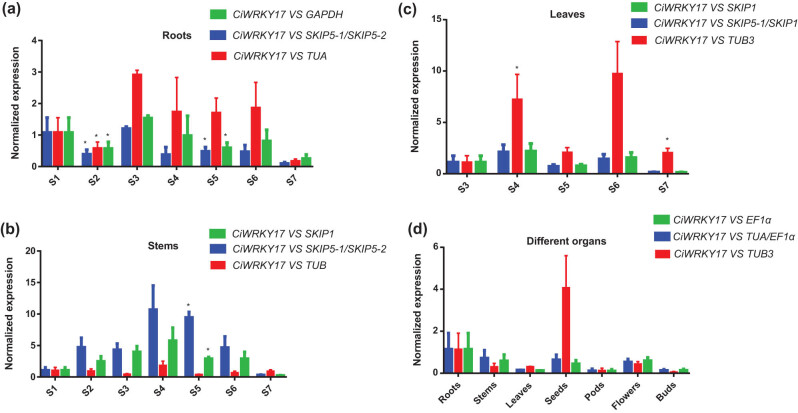
Effect of different RGs on normalization of the relative expression of *CiWRKY17*: (a) roots; (b) stems; and (c) leaves from different developmental stages; (d) different organs (buds collected from the stage of S3, flowers collected from S4, pods collected from S5, and roots, stems, leaves, and seeds collected from S6); (a–d) green, blue, and red represent the most stable RG, the most stable RG combination, and the worst RG, respectively; (a–d) represent samples containing three biological replicates (data were presented as means ± SE of three independent biological replicates; ^∗^ *p* < 0.05, ^∗∗^ *p* < 0.01, and ^∗∗∗^ *p* < 0.001, by Student’s *t*-test).

In order to validate the identified RGs and to demonstrate the application of the stable RGs selected by geNorm, NormFinder, BestKeeper, and RefFinder under the studied conditions, the transcript profile of *CiWRKY17*, which had been confirmed to express in leaf, root, and stem previously [4], was assayed again using the RGs (including the least and most stable RGs and their combinations). As shown in Figure 7, using the 2^−ΔΔCt^ method, the expression level of *CiWRKY17* was similar when we used the most stable RG and RG combination to normalize the RT-qPCR data. However, the results varied using the least stable RG.

## 4 Discussion

The powerful technique RT-qPCR has been widely used for the detection and quantification of gene expression in plants. In order to interpret RT-qPCR data accurately and reliably, appropriate RGs are essential. Reports on several plant species, such as *Undaria pinnatifida* [25], *Raphanus sativus L.* [26], *Lycoris aurea* [27], *Lagerstroemia indica* and *L. speciosa* [28], *Eucommia ulmoides* Oliver [29], *Pyrus* L. [30], and *Davidia involucrata* Baill. [31], had shown the importance of validating appropriate RGs for normalizing RT-qPCR data. In addition, some studies have shown that the best RG was different for different samples of organs or experimental conditions [26,29,32]. RGs of *C. intermedia* [21] had been screened under various abiotic stresses but had not been screened in different organs collected from different developmental stages, which led to difficulties in the normalization of the development-related gene. Therefore, it is particularly important to screen RGs in different organs at various development stages.

Current studies showed no perfect analysis software to evaluate the stability of RGs. In order to obtain the stable RG, we selected four kinds of analysis software to evaluate. Among them, geNorm [18], NormFinder [19], and BestKeeper [20] obtained the best RGs, respectively: *CiEF1α*, *CiGAPDH*, and *CiSKIP1* in roots; *CiUBQ*, *CiSKIP1*, and *CiGAPDH* in stems; *CiSKIP5-2*, *CiEF1α*, and *CiCAP* in leaves; *CiUBQ*, *CiTUA*, and *CiSKIP1* in different organs; and *CiSKIP1*, *CiEF1α*, and *CiSKIP1* in all samples. The above results indicated that different analysis software indeed screened different RGs, which might be caused by the different algorithms of this software, and this might also be the case in other reports [29,33,34]. Since different software came to a diverse conclusions, in order to obtain the best RG, we chose RefFinder [22] to integrate these conclusions and finally concluded that the best RG was *CiGAPDH* in roots, *CiSKIP1* in stems, *CiSKIP1* in leaves, *CiTUB* in different organs, and *CiEF1α* in all samples. In addition, geNorm could obtain the most suitable number of RGs according to *V*_*n*_/*V*_*n*+1_ [18]. For example, *V*4/*V*5 was less than 0.15 in all samples, indicating that four RGs were the most suitable. However, considering that multiple RGs would make the experiment more complicated and time-consuming, 1–2 RG was suitable to normalize the target genes for convenient operation. Therefore, according to the analysis results of geNorm, the optimal combination of RGs was obtained: *SKIP5-1/SKIP5-2* in roots, *SKIP5-1/SKIP5-2* in stems, *SKIP5-1/SKIP1* in leaves, *TUA*/*EF1α* in different organs, and *TUB/EF1α* in all samples.

To sum up, we found that the optimal RGs varied in different organ samples, and the optimal combination of RGs was different except for root and stem. In addition, previous studies on the selection of plant RGs mainly focused on different hormones [11,35], stress treatments [29,36,37], and different tissues/organs [9,12,38], but there were no studies on the selection of plant RGs in different organs collected from various development stages. This study aimed to screen the RGs of different organs at different developmental periods. We also used the most and least stable RGs, and their combination to normalize the transcript of a known gene, *CiWRKY17*, to evaluate the practicality of the selected RGs, and the results indicated that the selected RGs were reliable.

## 5 Conclusion

In conclusion, the 11 RGs were systematically selected and evaluated using RT-qPCR in seven organ types and at seven different developmental stages, using geNorm, NormFinder, BestKeeper, and RefFinder software. Then the selected RGs were further validated by analysis of the *CiWRKY17* expression in different organs. And the best RGs and the best combination of RGs were obtained. This study will improve the accuracy of the RT-qPCR results and lay the foundation for future studies on development-related genes of *C. intermedia*.

## Supplementary Material

Supplementary Figure

## References

[1] Zhang H, Ming T, Hui C, Tian Z, Xue Y, Ye F. Communities of arbuscular mycorrhizal fungi and bacteria in the rhizosphere of Caragana korshinkii and Hippophae rhamnoides in Zhifanggou watershed. Plant Soil. 2010;326:415–24.

[2] He Y, Dong Y, Yang X, Guo D, Wang Q. Functional activation of a novel R2R3-MYB protein gene, GmMYB68, confers salt-alkali resistance in soybean (Glycine max L.). Genome. 2020;63(1):13–26. 10.1139/gen-2018-0132 31550433

[3] Huang R, Liu D, Huang M, Ma J, Li Z, Li M, et al. CpWRKY71, a WRKY transcription factor gene of wintersweet (Chimonanthus praecox), promotes flowering and leaf senescence in Arabidopsis. Int J Mol Sci. 2019;20:5325. 10.3390/ijms20215325 PMC6862124 31731556

[4] Wan Y, Mao M, Wan D, Yang Q, Yang F, Mandlaa G, et al. Identification of the WRKY gene family and functional analysis of two genes in Caragana intermedia. BMC Plant Biol. 2018;18:31. 10.1186/s12870-018-1235-3 PMC5807834 29426284

[5] Yu N, Yang JC, Yin GT, Li RS, Zou WT. Genome-wide characterization of the SPL gene family involved in the age development of Jatropha curcas. BMC Genomics. 2020;21:368. 10.1186/s12864-020-06776-8 PMC7238634 32434522

[6] Yuanyuan X, Xianwen Z, Yiqin G, Liang X, Yan W, Liwang L. Evaluation of reference genes for gene expression studies in radish (Raphanus sativus L.) using quantitative real-time PCR. Biochem Biophys Res Commun. 2012;424:398–403. 10.1016/j.bbrc.2012.06.119 22771808

[7] Bustin SA. Quantification of mRNA using real-time reverse transcription PCR (RT-PCR): trends and problems. J Mol Endocrinol. 2002;29:23–39. 10.1677/jme.0.0290023 12200227

[8] Bustin SA, Benes V, Nolan T, Pfaffl MW. Quantitative real-time RT-PCR – a perspective. J Mol Endocrinol. 2005;34:597–601. 10.1677/jme.1.01755 15956331

[9] Wang JJ, Han S, Yin W, Xia X, Liu C. Comparison of reliable reference genes following different hormone treatments by various algorithms for qRT-PCR analysis of metasequoia. Int J Mol Sci. 2018;20(1):34. 10.3390/ijms20010034 PMC6337471 30577651

[10] Song Y, Wang Y, Guo D, Jing L. Selection of reference genes for quantitative real-time PCR normalization in the plant pathogen Puccinia helianthi Schw. BMC Plant Biol. 2019;19:20. 10.1186/s12870-019-1629-x PMC6329156 30634896

[11] Yan H, Zhang Y, Xiong Y, Chen Q, Liang H, Niu M, et al. Selection and validation of novel RT-qPCR reference genes under hormonal stimuli and in different tissues of Santalum album. Sci Rep. 2018;8(1):17511. 10.1038/s41598-018-35883-6 PMC6269485 30504917

[12] Gao S, Wang G, Huang Z, Lei X, Bian Y, Liu Y, et al. Selection of reference genes for qRT-PCR analysis in lentinula edodes after hot-air drying. Molecules. 2018;24(1):136. 10.3390/molecules24010136 PMC6337709 30602709

[13] Wang X, Fu Y, Ban L, Wang Z, Feng G, Li J, et al. Selection of reliable reference genes for quantitative real-time RT-PCR in alfalfa. Genes Genet Syst. 2014;90:175–80. 10.1266/ggs.90.175 26510572

[14] Xie LH, Quan X, Zhang J, Yang YY, Sun RH, Xia MC, et al. Selection of reference genes for real-time quantitative PCR normalization in the process of Gaeumannomyces graminis var. tritici infecting wheat. Plant Pathol. 2019;35:11–8. 10.5423/PPJ.OA.03.2018.0038 PMC6385657 30828275

[15] Sadritdinova AF, Snezhkina AV, Dmitriev AA, Krasnov GS, Astakhova LN, Kudryavtsev AA, et al. A new reference gene, for quantitative real-time PCR assay of the starfish Asterias rubens pyloric ceca. Dokl Biol Sci. 2013;452:310–2. 10.1134/S0012496613050050 24150654

[16] Kuhn Klumb E, Neutzling Rickes L, Bolacel Braga EJ, Bianchi VJ. Evaluation of stability and validation of reference genes for real time PCR expression studies in leaves and roots of Prunus spp. rootstocks under flooding. Sci Hortic. 2019;247:310–9.

[17] Delporte M, Legrand G, Hilbert J-L, Gagneul D. Selection and validation of reference genes for quantitative real-time PCR analysis of gene expression in Cichorium intybus. Front Plant Sci. 2015;6:651. 10.3389/fpls.2015.00651 PMC4539468 26347767

[18] Vandesompele J, Preter KD, Pattyn F, Poppe B, Roy NV, Paepe AD, et al. Accurate normalization of real-time quantitative RT-PCR data by geometric averaging of multiple internal control genes. Genome Biol. 2002;3(7):1–2. 10.1186/gb-2002-3-7-research0034 PMC126239 12184808

[19] Andersen CL, Jensen JL, Ørntoft TF. Normalization of real-time quantitative reverse transcription-PCR data: a model-based variance estimation approach to identify genes suited for normalization, applied to bladder and colon cancer data sets. Cancer Res. 2004;64:5245–50. 10.1158/0008-5472.CAN-04-0496 15289330

[20] Pfaffl MW, Tichopad A, Prgomet C, Neuvians TP. Determination of stable housekeeping genes, differentially regulated target genes and sample integrity: BestKeeper–Excel-based tool using pairwise correlations. Biotechnol Lett. 2004;26:509–15. 10.1023/b:bile.0000019559.84305.47 15127793

[21] Zhu J, Zhang L, Li W, Han S, Yang W, Qi L. Reference gene selection for quantitative real-time PCR normalization in Caragana intermedia under different abiotic stress conditions. PLoS One. 2013;8(1):e53196. 10.1371/journal.pone.0053196 PMC3534648 23301042

[22] Yang Q, Yin J, Li G, Qi L, Yang F, Wang R, et al. Reference gene selection for qRT-PCR in Caragana korshinskii Kom. under different stress conditions. Mol Biol Rep. 2014;41:2325–34. 10.1007/s11033-014-3086-9 24452712

[23] Xie F, Peng X, Chen D, Xu L, Zhang B. miRDeepFinder: a miRNA analysis tool for deep sequencing of plant small RNAs. Plant Mol Biol. 2012;80:75–84. 10.1007/s11103-012-9885-2 22290409

[24] Hellemans J, Mortier G, Paepe AD, Speleman F. qBase relative quantification framework and software for management and automated analysis of real-time quantitative PCR data. Genome Biol. 2007;8(2):R19. 10.1186/gb-2007-8-2-r19 PMC1852402 17291332

[25] Li J, Huang H, Shan T, Pang S. Selection of reference genes for real-time RT-PCR normalization in brown alga Undaria pinnatifida. J Appl Phycol. 2018;31:787–93.

[26] Karanja BK, Fan L, Xu L, Wang Y, Zhu X, Tang M, et al. Genome-wide characterization of the WRKY gene family in radish (Raphanus sativus L.) reveals its critical functions under different abiotic stresses. Plant Cell Rep. 2017;36:1757–73. 10.1007/s00299-017-2190-4 28819820

[27] Ma R, Xu S, Zhao Y, Xia B, Wang R. Selection and validation of appropriate reference genes for quantitative real-time PCR analysis of gene expression in Lycoris aurea. Front Plant Sci. 2016;7:536. 10.3389/fpls.2016.00536 PMC4843812 27200013

[28] Zheng T, Chen Z, Ju Y, Zhang H, Cai M, Pan H, et al. Reference gene selection for qRT-PCR analysis of flower development in Lagerstroemia indica and L. speciosa. PLoS One. 2018;13:e0195004. 10.1371/journal.pone.0195004 PMC5868847 29579116

[29] Ye J, Jin CF, Li N, Liu MH, Fei ZX, Dong LZ, et al. Selection of suitable reference genes for qRT-PCR normalisation under different experimental conditions in Eucommia ulmoides Oliv. Sci Rep. 2018;8:15043. 10.1038/s41598-018-33342-w PMC6177395 30301911

[30] Han JC, Xu F, Du J, Zhang YJ, Wei YJ, Li HB, et al. Systematic selection and validation of appropriate reference genes for gene expression studies by quantitative real-time PCR in pear. Acta Physiol Plant. 2015;37:40–301.

[31] Ren R, Huang F, Gao R, Dong X, Peng J, Cao F, et al. Selection and validation of suitable reference genes for RT-qPCR analysis in dove tree (Davidia involucrata Baill.). Trees. 2019;33:837–49.

[32] Zheng L, Ge XX, Wu XM, Kou SJ, Chai LJ, Guo WW. Selection and validation of suitable reference genes for mRNA qRT-PCR analysis using somatic embryogenic cultures, floral and vegetative tissues in citrus. Plant Cell Tissue Organ. 2013;113:469–81.

[33] Tian C, Jiang Q, Wang F, Wang GL, Xu ZS, Xiong AS. Selection of suitable reference genes for qPCR normalization under abiotic stresses and hormone stimuli in carrot leaves. PLoS One. 2015;10:e0117569. 10.1371/journal.pone.0117569 PMC4319972 25658122

[34] Li W, Lihui Z, Yandi Z, Guodong W, Dangyu S, Yanwen Z. Selection and validation of appropriate reference genes for quantitative real-time PCR normalization in staminate and perfect flowers of andromonoecious Taihangia rupestris. Front Plant Sci. 2017;8:729. 10.3389/fpls.2017.00729 PMC5437146 28579993

[35] Zhang Z, Li C, Zhang J, Chen F, Gong Y, Li Y, et al. Selection of the reference gene for expression normalization in Papaver somniferum L. under abiotic stress and hormone treatment. Genes. 2020;11(2):124. 10.3390/genes11020124 PMC7074096 31979407

[36] Jatav PK, Sharma A, Dahiya DK, Khan A, Agarwal A, Kothari SL, et al. Identification of suitable internal control genes for transcriptional studies in Eleusine coracana under different abiotic stress conditions. Physiol Mol Biol Plant. 2018;24:793–807. 10.1007/s12298-018-0544-1 PMC6103957 30150855

[37] Rapacz M, Stępień A, Skorupa K. Internal standards for quantitative RT-PCR studies of gene expression under drought treatment in barley (Hordeum vulgare L.): the effects of developmental stage and leaf age. Acta Physiol Plant. 2012;34:1723–33.

[38] Iskandar HM, Simpson RS, Casu RE, Bonnett GD, Maclean DJ, Manners JM. Comparison of reference genes for quantitative real-time polymerase chain reaction analysis of gene expression in sugarcane. Plant Mol Biol Rep. 2004;22:325–37.

