# Supplementary material for: Selection and validation of reference genes for RT-qPCR analysis of different organs at various development stages in Caragana intermedia
Source: Open Life Sci. 2022 Sep 14;17(1):1155–64. doi: 10.1515/biol-2022-0463 (PMC9483831; doi:10.1515/biol-2022-0463)
Supplement: Supplementary Figure [file biol-2022-0463-sm.pdf]

Supplementary material

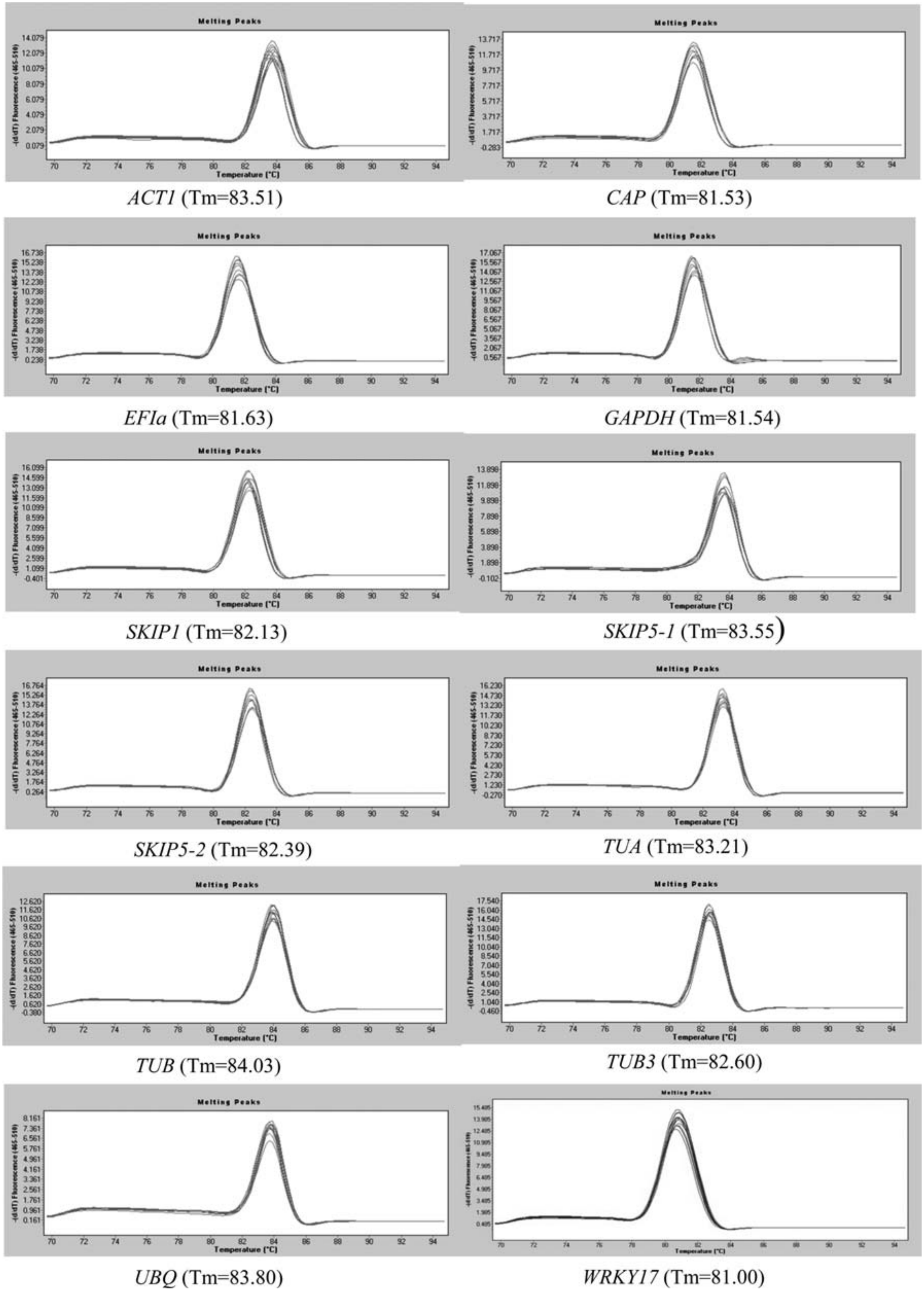

Figure S1: The melting curve of the 11 reference genes and *WRKY17*.

Table S1: The primers sequence, amplification efficiency, regression coefficient, and R2 value of this study: primer sequences refer to [1,2]

Gene name	Forward primer (5′–3′)	Reverse primer (5′–3′)	Efficiency	Regression coefficient	R^2
ACT	TGACAGGATGAGCAAGGAGA	GTTGGAAGGTGCTGAGGGA	1.0799	−3.1442	0.9883
CAP	GCATTCCGTGTACCTACTCCCA	CAACATCCTCGTCTGTGTATCCA	0.9987	−3.6158	0.9958
EF1α	ATTCTAAGAGGGCATTTGTCCAC	TTCATCATCACCAACACCATCAC	1.0071	−3.3305	0.9966
GAPDH	TTACAATGAGGCTTCTGGTGGA	AGACGAAGTTATCAGGGCGAA	1.0620	−3.1817	0.9629
SKIP1	AAAAAAGGGTACAAGAGTTGGACAAGG	TAACCATAACAGTCGGGGGAAC	0.9545	−3.4361	0.9928
SKIP5-1	TCAGGTGTGTCATTCTTTGGGTG	GGTGAACAGAGAGAGTGGCATTG	1.0049	−3.3103	0.9997
SKIP5-2	GCTTCCTTCCCCCTATTCCAG	AAAACACCAGGTTCAGATAAGTCTTTG	1.0396	−3.2305	0.9872
TUA	GTATTGAAGGAGGTGCGAAAGC	AAACAACAAATGATAATAACACATAGGC	0.9625	−3.4153	0.9988
TUB	GGGTGTCCTAACCTTGAGTTCTTG	CATATAGTCTCCAATGTCCATACCTTTC	0.9291	−3.5043	0.9995
TUB3	GGGAAGGATTGCGGTATGAGTA	TCGACATGATTTGGGTCTTTGTT	0.9860	−3.3559	0.9981
UBQ	CAAAAAGTCCCCTCGTTGTCTC	AGCAATCGTTCTTCCTAATGATCTAA	0.9217	−3.5251	0.9984
WRKY17	CAACCCAACAACCACTTAAACAC	GGTTCGGTTGAGTAAGGAGATGA	1.0867	−3.1303	0.9997

References

[1] Yang Q, Yin J, Li G, Qi L, Yang F, Wang R, et al. Reference gene selection for qRT-PCR in Caragana korshinskii Kom. under different stress conditions. Mol Biol Rep. 2014;41(4):2325–34.

[2] Wan Y, Mao M, Wan D, Yang Q, Yang F, Li G, et al. Identification of the WRKY gene family and functional analysis of two genes in Caragana intermedia. BMC Plant Biol. 2018;18(1):31.
